# An association between heart rate variability and incident heart failure in an elderly cohort

**DOI:** 10.1002/clc.24241

**Published:** 2024-02-25

**Authors:** Bozena Ostrowska, Lars Lind, Carina Blomström‐Lundqvist

**Affiliations:** ^1^ Department of Medical Sciences Uppsala University Uppsala Sweden; ^2^ School of Medical Science, Faculty of Medicine and Health Örebro University Örebro Sweden

**Keywords:** autonomic dysfunction, frequency domain, heart rate variability and heart failure, incident heart failure, LH/HF ratio, prediction model

## Abstract

**Background:**

Early identification of individuals at risk of developing heart failure (HF) may improve poor prognosis. A dominant sympathetic activity is common in HF and associated with worse outcomes; however, less is known about the autonomic balance before HF.

**Hypothesis:**

A low frequency/high frequency (L‐F/H‐F) ratio, index of heart rate variability, and marker of the autonomic balance predict the development of HF and may improve the performance of the HF prediction model when added to traditional cardiovascular (CV) risk factors.

**Methods:**

Individuals in the PIVUS (Prospective Investigation of the Vasculature in Uppsala Seniors) study (*n* = 1016, all aged 70 years) were included. Exclusion criteria were prevalent HF, electrocardiographic QRS duration ≥130 millisecond, major arrhythmias, or conduction blocks at baseline. The association between the L‐F/H‐F ratio and incident HF was assessed using Cox proportional hazard analysis. The C‐statistic evaluated whether adding the L‐F/H‐F‐ratio to traditional CV risk factors improved the discrimination of incident HF.

**Results:**

HF developed in 107/836 study participants during 15 years of follow‐up. A nonlinear, inverse association between the L‐F/H‐F ratio and incident HF was mainly driven by an L‐F/H‐F ratio of <30. The association curve was flat for higher values (hazard ratio, HR for the total curve = 0.78 [95% confidence interval, CI: 0.69−0.88, *p* < .001]; HR = 2 for L‐F/H‐F ratio = 10). The traditional prediction model improved by 3.3% (*p* < .03) when the L‐F/H‐F ratio was added.

**Conclusions:**

An L‐F/H‐F ratio of <30 was related to incident HF and improved HF prediction when added to traditional CV risk factors.

## Graphical abstract

Heart failure (HF) is characterized by autonomic dysfunction. Less is known about the autonomic balance before HF. This study tested the association between low frequency/high frequency (LF/HF) ratio and incident HF. The ratio <30 was related to incident HF and improved HF prediction when added to traditional risk factors.

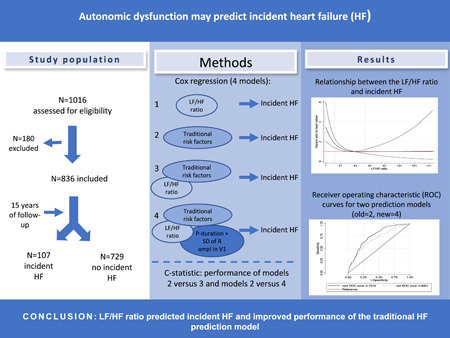

## 1 INTRODUCTION

Heart failure (HF) has a prevalence of 1%–2% in the general population, increasing to over 10% in individuals aged ≥70 years^1^ and is expected to increase because of the steadily increasing age of the population.^2^ Although substantial progress has been made in diagnosing and managing HF, the prognosis is still poor.^1^ The current definition of HF requires manifest clinical symptoms due to structural or functional cardiac abnormalities.^3^ Early detection of individuals at high risk of developing HF may enable targeted prevention and treatment and potentially improve the prognosis.^3^, ^4^, ^5^

HF is considered a disease with an autonomic imbalance.^6^ The persistent activation of the sympathetic nervous system (SNS) leads to a subsequent downregulation of beta‐receptors, which, being initially a compensatory mechanism to the hemodynamic changes in HF, contributes to further deterioration in the pump function and maladaptive cardiac remodeling.^7^, ^8^ An increased sympathetic drive in patients with manifest HF has been associated with adverse prognosis and lower functional capacity.^9^ Moreover, systemic inflammation has been recognized as a pathophysiological mechanism in HF.^10^, ^11^ A predominant sympathetic activity has also been associated with increased inflammation,^7^ and elevated C‐reactive protein (CRP) levels are highly prevalent in HF patients regardless of type.^11^ Heart rate variability (HRV) is a simple, noninvasive assessment of the autonomic function. It can be measured in time‐domain or frequency‐domain indices, assigning bands of high, low, or very low frequency (H‐F, L‐F, VL‐F) by power spectral analysis.^12^

We previously observed that a short P‐wave duration (Pdur) in lead V1 and an increased standard deviation of the R‐wave amplitude (SD Ramp) in V1 (study submitted to ESCHF ref. ESCHF‐23‐00374) were significantly associated with incident HF in the elderly.^13^ An increased level of relaxin that can shorten the Pdur^14^ as well as an enhanced SNS tone could explain these associations.

Therefore, the study's primary aim was to test whether an autonomic imbalance measured by the low frequency/high frequency (L‐F/H‐F) ratio preceded the development of HF and whether the discrimination of incident HF increased after adding the L‐F/H‐F ratio to traditional cardiovascular (CV) risk factors. A secondary aim was to evaluate whether the Pdur and the SD Ramp in lead V1 were related to the L‐F/H‐F ratio or the inflammatory marker C‐reactive protein (CRP).

## 2 METHODS

### 2.1 Study population

The present study was based on the PIVUS (Prospective Investigation of the Vasculature in Uppsala Seniors) study, which started in 2001 and invited individuals (*n* = 2025) aged 70 years from Uppsala, Sweden to participate.^15^ Of the 2025 invited subjects, 1016 (50% women) consented to enter the study.

In the present analysis, 836 PIVUS participants were included after excluding those with a prevalent diagnosis of HF, implanted pacemaker/defibrillator, atrial arrhythmias, second‐ and third‐degree atrioventricular block, delta waves, and QRS duration ≥130 millisecond.

### 2.2 Study design

In the PIVUS study, medical history and medications were recorded. A CV examination was performed at baseline, including blood pressure, electrocardiography (ECG), echocardiographic examination, and blood sampling for biomarkers. The six precordial ECG leads (V1 through V6) were recorded digitally for 5 minutes in the supine position and controlled breathing (12/min). The ECG was then analyzed by semiautomatic EClysis (AstraZeneca R&D) software with proven high reliability.^16^, ^17^ The positions of onsets and offsets of the P‐waves were initially determined by the Eclysis using unfiltered signals from the continuous ECG recording at 500 Hz and then evaluated by an experienced cardiologist, who was able to adjust the indicated markers.^17^ The RR interval durations were measured on the recorded ECG. The HRV analysis in the frequency domain was performed by proprietary software (Ekman Biomedical Data AB; [using MATLAB; Math‐Works Inc.]). A parametric autoregressive technique was used to calculate the HRV indices. The power of the variability of the normal‐to‐normal RR intervals was calculated in the H‐F band (0.15−0.25 Hz) and the L‐F band (0.03−0.15 Hz) and finally the L‐F/H‐F ratio was computed.

All examinations were repeated at the age of 75 and 80 years, except for the ECG analysis, which was limited to assessing whether atrial fibrillation (AF) was present. A very first clinical diagnosis of HF, defined in accordance with the concomitant ESC HF guidelines, was considered an incident HF. Data on HF and myocardial infarction (MI) diagnoses of the PIVUS study participants were retrieved from the Swedish Cause of Death Register and the Swedish Hospital Discharge Register, both with a high quality and accuracy.^13^ All retrieved diagnoses were then validated by an experienced cardiologist regarding their conformance to the guidelines. A detailed description of the PIVUS study has been published elsewhere.^13^, ^15^, ^17^

The Ethics Committee of the University of Uppsala approved the entire PIVUS study. A written informed consent was obtained from each participant before the start of the study.^18^

### 2.3 Statistical methods

The association between the L‐F/H‐F ratio and incident HF was assessed using the Cox proportional hazard model. Because of the theoretical possibility of a nonlinear relationship, the L‐H/F‐H ratio was modeled as a restricted cubic spline function with three knots (10th, 50th, and 90th percentile). An adjustment was made for sex, RR interval (age same in all individuals), beta‐blocking agents, systolic blood pressure, body mass index (BMI), and smoking. Lipids and diabetes were unrelated to incident HF in initial models for confounders.

In the next step, the associations between the L‐F and H‐F power as independent variables and incident HF were assessed in two Cox proportional hazard models with similar adjustment as in the previous analysis.

C‐statistic based on logistic regression was used to evaluate any improvement in HF discrimination after the addition of the L‐F/H‐F ratio to the traditional CV risk factors, including sex, systolic blood pressure, use of antihypertensive treatment, smoking, diabetes, BMI, and LDL and HDL cholesterol.^19^ Because age was the same in all subjects, this covariate was not included in the model.

In the next step, C‐statistic based on logistic regression was used to evaluate any improvement in HF discrimination after the addition of the L‐F/H‐F ratio along with Pdur in lead V1 and SD Ramp in lead V1 to the traditional CV risk factors.

All previous analyses were repeated with additional adjustments for AF with the onset after baseline and MI occurring after baseline.

Pairwise associations between the L‐F/H‐F ratio and the two ECG variables: the Pdur in lead V1 and the SD Ramp in lead V1, were evaluated using Spearman's correlation method.

Finally, Spearman's correlation assessed three pairwise correlations between the baseline level of the inflammatory marker CRP and L‐F/H‐F ratio, Pdur in lead V1, and SD Ramp in lead V1.

Possible differences in distribution of the L‐F/H‐F ratio, L‐F power, and H‐F power between subjects who developed HF and subjects who did not, were analyzed using the two‐sided Wilcoxon rank‐sum test and visualized by box plots.

## 3 RESULTS

Of 836 study participants (Table 1), 107 (13%) developed HF during a follow‐up of 15 years. During the follow‐up, AF developed in 148/836 (17.7%) subjects and 92/836 (11%) of subjects suffered from MI.

**Table 1:** Baseline characteristics in 836 individuals.

Variables	Values
Female sex, *n* (%)	418 (50)
Smoker, *n* (%)	84 (10)
BMI (kg/m^2^)	27 (4.2)
Beta‐blockers therapy, *n* (%)	170 (20)
Digitalis therapy, *n* (%)	4 (0.5)
ACEI or/and ARB therapy, *n* (%)	119 (14.2)
Systolic blood pressure (mmHg)	150 (22)
Use of antihypertensive treatment, *n* (%)	250 (30)
Diabetes mellitus, *n* (%)	95 (11)
LVEF	67 (6)
CRP (mg/L)	2.4 (5.1)
LDL (mmol/L)	3.4 (0.9)
HDL (mmol/L)	1.5 (0.4)
RR‐interval (ms)	980 (136)
L‐F (ms^2^) (median, IQR)	1667 (425−4474)
H‐F (ms^2^) (median, IQR)	146 (65−303)
L‐F/H‐F ratio (median, IQR)	13.6 (3.2−31.3)
Pdur in V1 (ms)	71.3 (20.9)
SD Ramp in V1 (mV) (median, IQR)	0.01 (0.006−0.013)

*Note*: The numbers represent the mean and standard deviation (SD) in brackets unless otherwise stated.

Abbreviations: ACEI, angiotensin‐converting enzyme inhibitors; ARB, angiotensin receptor blockers; BMI, body mass index; CRP, C‐reactive protein; HDL, high‐density lipoproteins; H‐F, high frequency; IQR, interquartile range; LDL, low‐density lipoproteins; L‐F, low frequency; LVEF, left ventricular ejection fraction; Pdur, P‐wave duration; Ramp, R‐wave amplitude; SD, standard deviation.

### 3.1 Association between HRV indices, EGG variables, and incident HF

**Figure 1:**
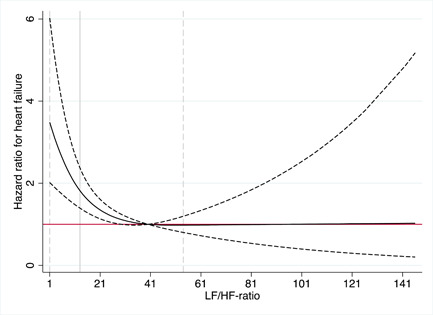
Relationship between the L‐F/H‐F ratio and incident heart failure (HF). The solid line represents the hazard ratio (HR) for incident HF. The dashed line represents the 95% confidence interval. The horizontal solid line represents the HR = 1. H‐F, high frequency power; L‐F, low frequency power.

In a Cox regression model a highly significant, nonlinear and inverse association was found between the low L‐F/H‐F ratio and incident HF following the adjustment for traditional CV risk factors and after the additional adjustment for AF and MI occurring after baseline. As indicated in Figure 1, the inverse relationship between the L‐F/H‐F ratio and incident HF was mainly found in individuals with an L‐F/H‐F ratio <30. At the same time, the association curve was flat for the higher values (hazard ratio [HR] for the total curve = 0.78 [95% confidence interval, CI: 0.69−0.88, *p* < .001]). The L‐F/H‐F ratio = 10 resulted in an HR of 2.0.

The relationship between the L‐F/H‐F ratio and incident HF was mainly due to the association between the low L‐F power and incident HF (HR: 0.78, 95% CI: 0.69−0.87, *p* < .001), as the H‐F power was not significantly associated with incident HF (HR: 0.96, 95% CI: 0.82−1.13, *p* = .65). There was no evidence for a difference in the distribution of the H‐F power between individuals who developed HF and individuals who did not (*p* = .66, two sample Wilcoxon rank‐sum test) (Table 2).

**Table 2:** Frequency domain indices in 836 individuals at baseline.

Variables	Incident HF			No incident HF		
	*N* = 107			*N* = 729		
	L‐F/H‐F ratio	L‐F power (ms^2^)	H‐F power (ms^2^)	L‐F/H‐F ratio	L‐F power (ms^2^)	H‐F power (ms^2^)
Median	6.08	1000.0	168.0	14.24	1737.5	145.5
IQR	1.36−24.41	193.0−3553.0	55.0−303.0	3.68−32.43	518.0−4600	65.0−305.0
Skewness	3.46	4.1	7.1	2.19	16.6	6.0
Probability (Wilcoxon)	0.0007	0.0008	0.99	0.0007	0.0008	0.99

*Note*: Three Wilcoxon rank‐sum tests compared indices of the same category (L‐F/H‐F ratio, L‐F power, and H‐F power) between individuals with incident HF and individuals without incident HF, respectively.

Abbreviations: HF, heart failure; H‐F, high frequency; IQR, interquartile range; L‐F, low frequency; ms, millisecond.

The two‐sample Wilcoxon sum‐rank test comparing the HF power between subjects with the LF/HF ratio >30 and subjects with the ratio <30, resulted in *p* < .0000, which implied a significant difference.

In addition, individuals with the ratio >30 had lower mean of the HF power compared to subjects with the ratio <30 (135.38 vs. 351.97, respectively) as well as lower maximal value of the HF power in the fourth quartile (2494 vs. 7783, respectively).

### 3.2 Discrimination of HF

Compared to the model with traditional CV risk factors alone, the addition of the L‐F/H‐F‐ratio factors improved HF predictive performance by 3.3% (*p* = .03) (AUROC curve = 0.69 with 95% CI = 0.63−0.74 vs. AUROC curve = 0.72 with 95% CI = 0.67−0.77). A similar improvement in C‐statistics was seen when the additional adjustment was made for MI (*n* = 92, *p* = .03). In contrast, there was no significant improvement after the additional adjustment for AF (*n* = 71, *p* = .08).

**Figure 2:**
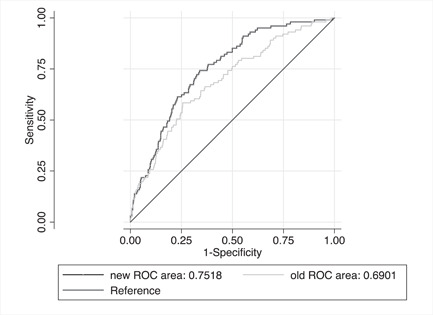
Receiver operating characteristic (ROC) curves for two models. “New” denotes a logistic regression model including traditional cardiovascular (CV) risk factors for heart failure (HF) plus the P‐wave duration in lead V1, the standard deviation of the R‐wave amplitude in lead V1 and the low‐frequency/high‐frequency ratio (L‐F/H‐F ratio). “Old” denotes a logistic regression model including traditional CV risk factors for HF.

Compared to the model with traditional CV risk factors alone, the addition of three variables (the Pdur in V1, the SD Ramp in lead V1, and the L‐F/H‐F ratio) further improved predictive performance by 6.1% (*p* = .0015) (AUROC curve = 0.69 with 95% CI = 0.63−0.74, AUROC curve = 0.75 with 95% CI = 0.70−0.80) (Figure 2). This improvement was still significant after the additional adjustment for MI (*p* = .01) and AF (*p* = .01).

### 3.3 Relationships between HRV variables, ECG variables, and CRP (Table 3)

**Table 3:** Correlations between the P‐wave duration in V1, the standard deviation of the R‐wave amplitude in V1, and the L‐F/H‐F ratio.

	CRP	L‐F/H‐F ratio	P‐wave duration	SD of Ramp
CRP	1.0			
L‐F/H‐F ratio	−0.013 (0.80)	1.0		
P‐wave duration	−0.025 (0.56)	0.174 (<0.001)	1.0	
SD of Ramp	0.038 (0.31)	−0.144 (<0.001)	−0.185 (<0.001)	1.0

Abbreviations: CRP, C‐reactive protein; H‐F, high frequency power; L‐F, low frequency power; Ramp, R‐wave amplitude SD, standard deviation.

Figures are Spearman's correlation coefficient + *p*‐value in brackets.

P‐wave duration and SD of the R‐wave amplitude were measured in lead V1.

Weak correlations were detected between the L‐H/F‐H ratio and the Pdur in V1 and between the L‐H/F‐H ratio and the SD Ramp in V1.^20^

Negligible pairwise correlations were observed between the baseline CRP level and the L‐H/F‐H ratio, the Pdur in V1, and the SD Ramp in V1.

## 4 DISCUSSION

The main finding of this study was the strong relationship between a low L‐F/H‐F ratio, which was consistent with an autonomic imbalance, and incident HF in an elderly population.

Ample evidence shows that elevated sympathetic activity and parasympathetic withdrawal^21^ characterize chronic HF. A decreased HRV in chronic HF is commonly known and associated with adverse outcomes.^21^ However, data on the relationship between HRV and incident HF are scarce, although the link between the time‐domain HRV indices and incident HF has been described.^22^, ^23^, ^24^ Our understanding is that the relationship between HRV in the frequency domain and incident HF is obscured.

The HRV spectral analysis in the frequency domain gives the distribution of the HRV power as a function of frequency: the H‐F component is thought to be mediated primarily by the vagal tone and the L‐F component through sympathetic and parasympathetic tone.^25^ Some authors have interpreted an increased L‐F power as high sympathetic activity^26^; others found an attenuation or absence of the L‐F component measured on short‐term ECG recordings in patients with severe HF, known to have a grossly elevated sympathetic tone.^27^ The decreased L‐F power in a chronic state of a high sympathetic drive may be due to a downregulation of the β‐adrenoceptors, which limits the responsiveness of the sinus node.^27^, ^28^ Another possible explanation is impaired central autonomic modulation or reduced baroreflex sensitivity.^21^ Moreover, the reduced L‐F power in chronic HF patients has been linked to the increasing severity of the disease and a higher risk of sudden death.^21^, ^29^

Some researchers have considered the L‐F/H‐F ratio to reflect the balance between the SNS and PNS activity.^25^, ^30^, ^31^ However, PNS and SNS interactions are complex and interdependent but not necessarily reciprocal or balanced,^28^ making the L‐F/H‐F ratio rather difficult to interpret concerning sympathetic or parasympathetic predominance.^32^ Low values of the L‐F/H‐F ratio have been associated with hyperglycemia and increased lifetime risk for CV disease.^6^, ^33^

In the present study a low L‐F/H‐F ratio was a strong predictor of incident HF. Therefore, we suggest that an autonomic imbalance precedes the development of HF. However, the primary mechanism of this finding is unclear, although the concomitant strong correlation between the reduced L‐F power and incident HF may indicate a sympathetic predominance before HF, possibly because of a dysregulation of baroreflex sensitivity. In addition, the present study found an increased H‐F power in individuals with higher risk for incident HF, which may argue against a reduced vagal activity as a main cause of the autonomic imbalance before HF.

This study adjusted for traditional CV risk factors, and an additional adjustment was made for AF and MI. Adding AF to the set of conventional CV risk factors in the C‐statistics analyses reduced the ability of the L‐F/H‐F ratio to improve discrimination. Because an autonomic imbalance increases the risk of AF^34^ and subsequently of HF, the AF in the present study should be considered an effect modifier rather than a confounder.

The optimal technique for HRV measurement has not yet been determined,^8^ but the frequency‐domain technique may be more appropriate for quantifying HRV compared to the time‐domain technique.^12^ Moreover, the time‐domain analysis is preferably performed from a long‐term recording. In contrast, the frequency‐domain analysis should be performed from a short‐term, standardized recording, which is more straightforward and potentially more applicable in clinical practice.^29^, ^35^

Although a large number of prediction models for incident HF have been developed,^36^ the traditional CV risk factors have remained the best predictors of HF.^19^ Some ECG parameters (left bundle branch block, ST‐segment depression, and left ventricular hypertrophy), as well as biomarkers (CRP, cardiac troponin, and N‐terminal pro‐brain natriuretic peptide), have also been proposed as predictors of incident HF.^19^, ^37^, ^38^, ^39^ However, incorporating ECG variables into different CV risk prediction models has resulted in only a 0.1%−5% increase in the discrimination capacity.^40^ The addition of the L‐F/H‐F ratio in our study, along with the Pdur and the SD Ramp in lead V1, improved the performance of the traditional HF prediction model by 6.1%, which should be considered a sizable improvement in comparison.^40^

Although sympathetic overactivity is linked to systemic inflammation in HF,^10^ we found no significant association between the baseline level of CRP and the two markers of the ANS balance: L‐F/H‐F ratio and the SD Ramp in V1.

The major strength of this study was the extended follow‐up, which enabled the detection of the majority of new HF cases. Another strength was the ECG analysis performed by the validated software and the complete data set at baseline.

Because data on left ventricular ejection fraction (EF) were not available at the time of HF diagnosis in most individuals, the distinction between HF with preserved EF and reduced EF was not possible, which is a limitation.

In conclusion, a decreased L‐F/H‐F ratio predicted incident HF in the elderly. Moreover, adding the L‐F/H‐F ratio to traditional risk factors improved the performance of the HF prediction model by 3.3%, which may be helpful in clinical practice. Although a 5‐minute ECG is seldom recorded in routine clinical practice, it is a widespread and feasible technique that could easily be adopted in primary care settings.

## CONFLICT OF INTEREST STATEMENT

Carina Blomstrom‐Lundqvist reports personal fees from Bayer, Medtronic, CathPrint, Philips, Sanofi Aventis, Boston Sci, Abbott, Milestone, Organon and Merck Sharp & Dohme outside the submitted work. The remaining authors declare no conflict of interest.

## Data Availability

The data that support the findings of this study are available on request from the corresponding author. The data are not publicly available due to privacy or ethical restrictions. Due to Swedish law and the Ethical Committee's permission, the data underlying this article cannot be shared publicly. The data may be shared based on a request to the corresponding author.
